# Prognostic value of regional lymph node involvement in patients with metastatic colorectal cancer: palliative versus curative resection

**DOI:** 10.1186/s12957-021-02260-z

**Published:** 2021-05-13

**Authors:** Ya-Ting Kuo, Wen-Sy Tsai, Hsin-Yuan Hung, Pao-Shiu Hsieh, Sum-Fu Chiang, Cheng-Chou Lai, Yih-Jong Chern, Yu-Jen Hsu, Jeng-Fu You

**Affiliations:** 1 Division of Colon and Rectal Surgery, Department of Surgery, Chang Gung Memorial Hospital at Linkou, Chang Gung University College of Medicine, No. 5, Fuxing Street, Guishan District, Taoyuan City, Taiwan; 2 Division of Colon and Rectal Surgery, Department of Surgery, New Taipei Municipal TuCheng Hospital, Chang Gung Memorial Hospital, New Taipei City, Taiwan

## Abstract

**Background:**

Approximately 20% of patients with colorectal cancer are initially diagnosed with stage IV disease. This study aims to examine the role of regional lymph node (LN) status in metastatic colorectal cancer (mCRC) with respect to clinicopathologic features and survival outcomes.

**Methods:**

We investigated 1147 patients diagnosed with mCRC and had undergone surgical resection of the primary CRC. A total of 167 patients were placed in the LN-negative (LN−) group and another 980 in the LN-positive (LN+) group.

**Results:**

LN+ patients exhibited a significantly higher rate of T4 tumors (p = 0.008), poorly differentiated adenocarcinoma (p < 0.001), lymphovascular invasion (p < 0.001), and perineural invasion (p < 0.001) than those in the LN− group. LN− patients had a significantly higher rate of lung metastasis (p < 0.001), whereas the rate of peritoneal seeding (p < 0.001) and systemic node metastasis (p < 0.001) was both significantly higher in the LN+ group. The 5-year overall survival (OS) in the LN+ group was significantly poorer than that in the LN− group (LN− vs. LN+ 23.2% vs. 18.1%; p = 0.040). In patients with curative resection, the 5-year OS rate has no significant difference between the two groups (LN− vs. LN+ 19.5% vs. 24.3%; p = 0.890).

**Conclusions:**

Metastatic CRC patients with LN+ who underwent primary tumor resection may present with more high-risk pathological features, more peritoneal seeding, and systemic node metastasis, but less lung metastasis than LN− patients. LN+ patients had poorer long-term outcomes compared with that in LN− patients. Nevertheless, with curative resection, LN+ patients could have similar survival outcomes as LN− patients.

## Introduction

Approximately 20% of newly diagnosed colorectal cancer patients present with synchronous distant metastasis, the majority of whom are only eligible for treatment with palliative intent, and the prognosis in these patients is usually poor [1–5]. In patients with metastatic colorectal cancer (mCRC), curative or palliative operation is sometimes performed and has favorable prognostic impact, especially when the primary tumor and metastatic region can be resected [6–11]. Advances in systemic chemotherapy and target therapy have provided therapeutic options and led to a remarkable increase in OS (from less than 1 year to 30 months or longer) [12–18]. The 5-year OS rate is approximately 20% in patients treated with chemotherapy alone [19, 20]. In patients with liver metastases, the 5-year OS rate after curative resection of both primary and metastatic lesions varies from 25 to 58%, whereas in those with lung metastases, it varies from 25 to 52% [21, 22].

Considering metastasis can be fatal, its development is a concern for patients and clinicians. Clinical data represent an essential basis for postoperative clinical surveillance in mCRC patients. However, the mechanisms and routes of metastasis in CRC are poorly understood, and the prognostic factors can only be surmised from demographic data and epidemiologic reports. According to previous reports, around 18% of patients with mCRC did not present regional LN involvement [23, 24]. A newly developed mouse model of CRC has demonstrated that liver metastases can develop without prior LN involvement [25].

The proposed metastatic routes are hematogenous, lymphatic, and transcoelomic/transperitoneal spread, and the common CRC metastatic sites are the liver, lung, peritoneum, and systemic LNs [26]. Since venous drainage of the intestinal tract occurs through the portal system, the first site of hematogenous spread is usually the liver, followed by the lungs, bones, brain, and other sites. However, the distal rectum may initially metastasize to the lungs because drainage return travels from the inferior rectal vein to the inferior vena cava (IVC) rather than the portal venous system. Tumor cells in some patients are directly transferred to the lungs through the lymphatic system, whereas others may have peritoneal seeding through transcoelomic/transperitoneal spread [26–28].

Metastatic patterns usually differ according to histologic type, histologic grade, and tumor location. According to Riihimäki et al., rectal cancer more frequently metastasized into the thoracic organs and nervous system than into the peritoneum, and more peritoneum seeding occurred in mucinous and signet ring adenocarcinoma. Survival in CRC patients with solitary metastases ranged from 5 to 19 months, depending on the T and N stage [29].

In this study, we examined the role of regional LN status in patients with mCRC concerning clinicopathologic features, metastatic sites, and survival outcomes. This information using a real-world dataset may help elucidate the association of regional LN and distant metastatic organs in patients with mCRC.

## Methods

### Patients and variables

Detailed data for patients who had been diagnosed with mCRC who had undergone primary colorectal tumor resection with or without metastasectomy between January 2003 and December 2015 were retrospectively retrieved from routinely collected data in the Colorectal Section Tumor Registry at Chang-Gung Memorial Hospital (CGMH). The hospital’s institutional review board approved this study. Clinical staging was determined mainly through computed tomography, whereas some peritoneal seeding tumors were confirmed during operation. Exclusion criteria included distant metastases of non-colorectal origin, patients receiving local excision or bypass surgery with no primary tumor resection, patients receiving neoadjuvant therapy, and patients undergoing emergency surgery. Patients with mCRC were then divided into two groups depending on whether regional LN metastases were present. Following primary tumor resection, patients whose pathologic reports indicated no regional LN metastasis were classified as the lymph node-negative (LN−) group, whereas those with any positive regional LN were classified as the lymph node-positive (LN+) group.

The available medical records included data on sex, age, body mass index (BMI), family cancer history (including familial adenomatous polyposis and hereditary nonpolyposis colorectal cancer), and underlying medical conditions (e.g., hypertension, cardiac disease, and diabetes mellitus). Preoperative blood tests were also recorded, including hemoglobin, albumin, creatinine, carcinoembryonic antigen (CEA), and total bilirubin levels. Tumor-related clinicopathologic variables included tumor location, diameter and invasion depth, number of positive LNs, circumferential involvement, curative resection, histologic type, histologic grade, desmoplastic reaction, tumor necrosis, lymphovascular invasion (LVI), and perineural invasion (PNI).

Treatment strategies, including postoperative palliative therapy (target therapy, chemotherapy, radiation, chemoradiation), were recorded. The selection of regimen for postoperative palliative therapy was independent to regional lymph node status. The choice of surgical removal for primary CRC was influenced by whether patients presented with symptoms such as tumor bleeding or obstruction, the potential for curative resection of the primary tumor and distant metastatic sites, and the individual physician’s judgment. Different physicians in the colorectal section of the Chang-Gung Memorial Hospital (CGMH) adopted similar treatment strategies, and all patients were assessed at weekly multidisciplinary team meetings to clarify the diagnosis and metastatic sites according to their clinical information and develop treatment plans. The presence of distant metastasis was documented, with metastatic site subgroups comprising the liver, lungs, peritoneal carcinomatosis, systemic node, ovary, bone, brain, and others (including rare locations, such as the bladder, uterus, pelvic wall, adrenal gland, skin, and kidney).

Morbidity and mortality were classified as postoperative complications. Morbidity was defined as wound-related (wound infection or dehiscence), pulmonary (atelectasis, pneumonia), cardiovascular (myocardial infarction, stroke, embolism), urinary (urinary tract infection, neurogenic bladder), gastrointestinal (obstruction, ileum, bleeding), anastomosis-related (leakage, stenosis), and other complications occurring within 30 days after surgery. Postoperative mortality was defined as death occurring during the hospital stay or within 30 days after surgery. Prognosis was evaluated based on OS, with the OS interval defined as the duration between the date of initial surgery and the date of death or the latest follow-up.

### Statistical analysis

All analyses were performed using IBM SPSS Statistics, version 24.0 (IBM Corp., Armonk, NY, USA). Clinicopathologic characteristics were compared using the chi-squared test for categorical variables, Student’s *t*-test for continuous data with normal distribution, and Mann-Whitney U test for continuous data against normal distribution. OS was calculated through univariate analyses using the Kaplan–Meier method. Differences were estimated using the log-rank test. Statistical significance was set at p < 0.05.

## Results

**Fig. 1:**
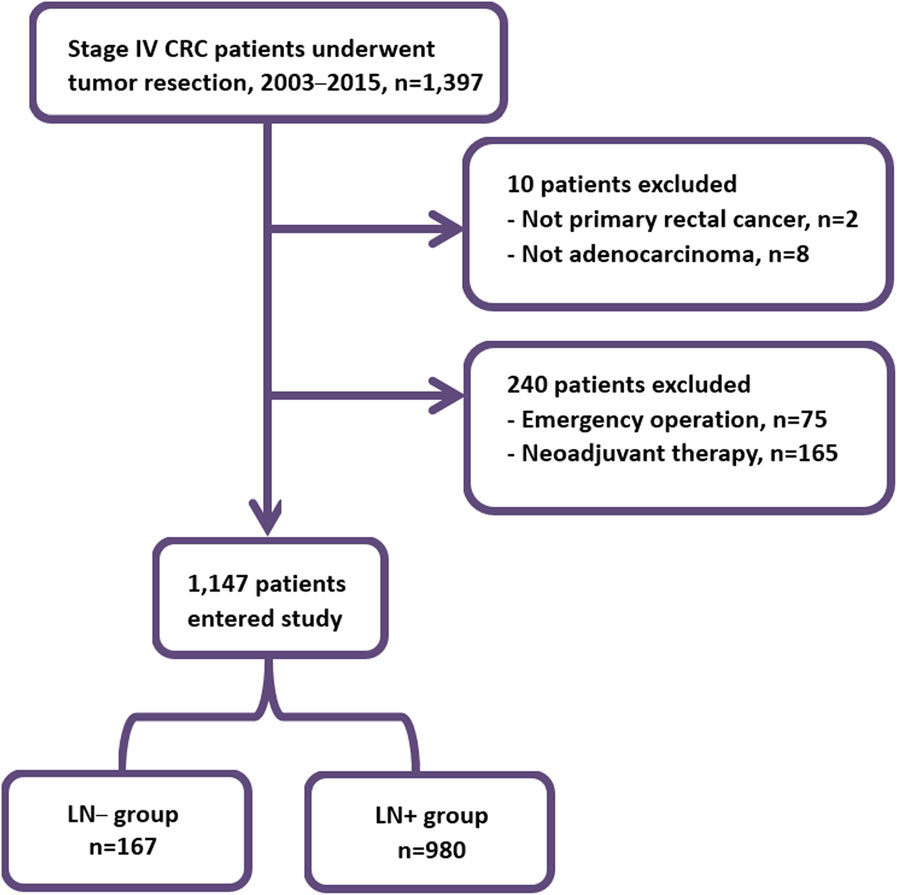
Flowchart of patient selection

We enrolled and analyzed 1147 patients, divided into the LN− group (167 patients) and LN+ group (980 patients) (Fig. 1). The mean age of these patients was 61.7 years, and their median follow-up time was 23.9 months.

Demographic data are listed in Table 1. No statistical differences were found between the two groups for sex, age, BMI, family cancer history, and presence of comorbidities, including hypertension, cardiac disease, and diabetes mellitus. No statistical differences were found between the two groups in preoperative laboratory data, including CEA, hemoglobin, albumin, creatinine, and total bilirubin levels.

**Table 1:** Demographics and laboratory variables

	LN− group n = 167 (%)	LN+ group n = 980 (%)	p value
Sex			
Male	93 (55.7)	556 (56.7)	0.801
Female	74 (44.3)	424 (43.3)	
Age (year)	62.0 ±15.1	61.7 ±14.2	0.776
Age <50	35 (21)	192 (19.6)	0.682
Age ≥50	132 (79)	788 (80.4)	
BMI^a^	23.4 ±3.9	23.5 ±3.6	0.738
<25	110 (66.7)	674 (69.6)	0.458
≥25	55 (33.3)	295 (30.4)	
Family cancer history			
Yes	58 (34.9)	387 (40.2)	0.198
No	108 (65.1)	575 (59.8)	
Medical illness			
Hypertension	52 (31.1)	308 (31.4)	0.940
Cardiac disease	10 (6)	73 (7.4)	0.501
Diabetes mellitus	26 (15.6)	149 (15.2)	0.904
CEA^a^ (ng/mL)			
CEA <5	48 (29.1)	263 (27.6)	0.687
CEA ≥5	117 (70.9)	691 (72.4)	
Hemoglobin (g/dL)	11.6 ± 2.4	11.6 ± 2.5	0.879
≥10	128 (76.6)	733 (74.8)	0.609
<10	39 (23.4)	247 (25.2)	
Albumin^a^ (g/dL)	3.9 ± 0.6	3.9 ± 0.6	0.793
Albumin <3.5	29 (18)	161 (17.1)	0.775
Albumin ≥3.5	132 (82)	781 (82.9)	
Creatinine^a^ (mg/dL)			
Normal	138 (83.6)	793 (82.9)	0.807
Abnormal	27 (16.4)	164 (17.1)	
Total bilirubin^a^ (mg/dL)			
Normal	152 (98.7)	853 (96.4)	0.136
Abnormal	2 (1.3%)	32 (3.6)	

*CEA* carcinoembryonic antigen

^a^Number of missing data: BMI 13, CEA 28, albumin 44, creatinine 25, total bilirubin 108

The treatment data are listed in Table 2. No significant difference was found between the two groups in terms of postoperative palliative therapy (p = 0.506), postoperative morbidity (p = 0.832), postoperative mortality (p = 0.632), and length of hospital stay (p = 0.145). A total of 178 patients (15.5%) had postoperative morbidities (25 in the LN− group, 153 in the LN+ group), and postoperative mortality occurred in 16 patients (1.4%; 3 in the LN− group, 13 in the LN+ group).

**Table 2:** Treatment modality and operative outcomes

	LN− group n = 167 (%)	LN+ group n = 980 (%)	p value
Postoperative palliative therapy			
Yes	135 (80.8)	814 (83.1)	0.506
No	32 (19.2)	166 (16.9)	
Morbidity			
Yes	25 (15)	153 (15.6)	0.832
No	142 (85)	827 (84.4)	
Mortality			
Yes	3 (1.8)	13 (1.3)	0.632
No	164 (98.2)	967 (98.7)	
Days of admission	14 [11–19]*	13 [11–17]*	0.145

*Median [25 percentile–75 percentile]

The pathological characteristics are displayed in Table 3. The tumor location distribution was similar in both groups (p = 0.066). No statistical differences between the two groups were found for circumferential tumor involvement (p = 0.631) and tumor size larger than 5 cm (p = 0.313). Patients in the LN+ group had more T4 tumors than those in the LN− group (percentage of patients with T4 tumors 48.2% vs. 37.1%, respectively; *p* = 0.008). Similarly, no statistical differences were found in the local tumor clearance rate of the two groups. The median positive LN count in the LN+ group was 6 (range, 1–84). Moreover, no statistical differences between the groups were found for histology type, desmoplastic tumor reaction, and tumor necrosis. In primary tumor histology grade, the LN+ group exhibited a higher rate of poorly differentiated adenocarcinoma than that in the LN− group (LN− vs. LN+ 7.2% vs. 19.2%; p < 0.001). LVI and PNI both occurred more frequently in the LN+ group than that in the LN− group (LVI rate in LN− vs. LN+, 23.4% vs. 77.4%, p < 0.001; PNI rate in LN− vs. LN+, 34.7% vs. 59.7%, p < 0.001).

**Table 3:** Tumor characteristics

	LN− group n = 167 (%)	LN+ group n = 980 (%)	p value
Tumor location			
Right side colon	34 (20.4)	284 (29)	0.066
Left side colon	71 (42.5)	360 (36.7)	
Rectum	62 (37.1)	336 (34.3)	
Circumferential involvement			
Yes	122 (73.1)	734 (74.9)	0.631
No	45 (26.9)	246 (25.1)	
Associated polyps			
Yes	61 (36.5)	394 (40.2)	0.369
No	106 (63.5)	586 (59.8)	
Tumor diameter (cm)			
< 5cm	82 (49.1)	437 (44.6)	0.313
≥ 5cm	85 (50.9)	543 (55.4)	
Depth of tumor invasion			
T4	62 (37.1)	472 (48.2)	0.008
Non-T4	105 (62.9)	508 (51.8)	
Examined LN number	25 [16–39]*	28 [19–39]*	0.038
Total positive LN number	0 [0–0]*	6 [3–11]*	<0.001
Local tumor clearance			
R0	59 (35.3)	283 (28.9)	0.092
Non-R0	108 (64.7)	697 (71.1)	
Histologic type			
Adenocarcinoma	155 (92.8)	873 (89.1)	0.121
Signet ring cell adenocarcinoma	1 (0.6)	34 (3.5)	
Mucinous adenocarcinoma	11 (6.6)	73 (7.4%)	
Histologic grade^a^			
Well or moderately differentiated	155 (92.8)	789 (80.8)	<0.001
Poorly differentiated	12 (7.2)	188 (19.2)	
Desmoplastic reaction^a^			
Mild	41 (24.6)	209 (21.3)	0.331
Moderate	106 (63.5)	613 (61.6)	
Marked	20 (12)	157 (16)	
Tumor necrosis^a^			
Mild	72 (49.7)	414 (47)	0.824
Moderate	53 (36.6)	344 (39.1)	
Marked	20 (13.8)	122 (13.9)	
Lymphovascular invasion^a^			
Yes	39 (23.4)	755 (77.4)	<0.001
No	128 (76.6)	221 (22.6)	
Perineural invasion^a^			
Yes	58 (34.7)	581 (59.7)	<0.001
No	109 (65.3)	393 (40.3)	

*Median [25 percentile–75 percentile]

^a^Number of missing data: histologic grade 3, desmoplastic reaction 1, tumor necrosis 122, lymphovascular invasion 4, perineural invasion 6

An analysis of the metastatic characteristics is presented in Table 4. The rate of liver metastasis was similar in both groups (p = 0.114). Patients in the LN− group had a higher lung metastasis rate than those in the LN+ group (25.7% vs. 14.6%, respectively; p < 0.001). The frequency of peritoneal seeding and systemic node metastasis was higher in the LN+ group (peritoneal seeding in LN− vs. LN+, 17.4% vs. 32.4%, p < 0.001; systemic node metastasis in LN− vs. LN+, 6% vs. 17%, p < 0.001). No statistical differences between the two groups were found in terms of the ovary, bone, brain, and other metastasis sites, which included the bladder, uterus, pelvic wall, adrenal gland, skin, and kidney.

**Table 4:** Metastatic sites

	LN− group n = 167 (%)	LN+ group n = 980 (%)	p value
Liver	106 (63.5)	558 (56.9)	0.114
Lung	43 (25.7)	143 (14.6)	<0.001
Peritoneal seeding	29 (17.4)	318 (32.4)	<0.001
Systemic node	10 (6)	167 (17)	<0.001
Ovary	6 (3.6)	53 (5.4)	0.326
Bone	2 (1.2)	7 (0.7)	0.513
Brain	0 (0)	2 (0.2)	0.559
Other metastatic sites^a^	6 (3.6)	44 (4.5)	0.600

^a^Other metastatic sites include the bladder, uterus, pelvic wall, adrenal gland, skin, and kidney

**Fig. 2:**
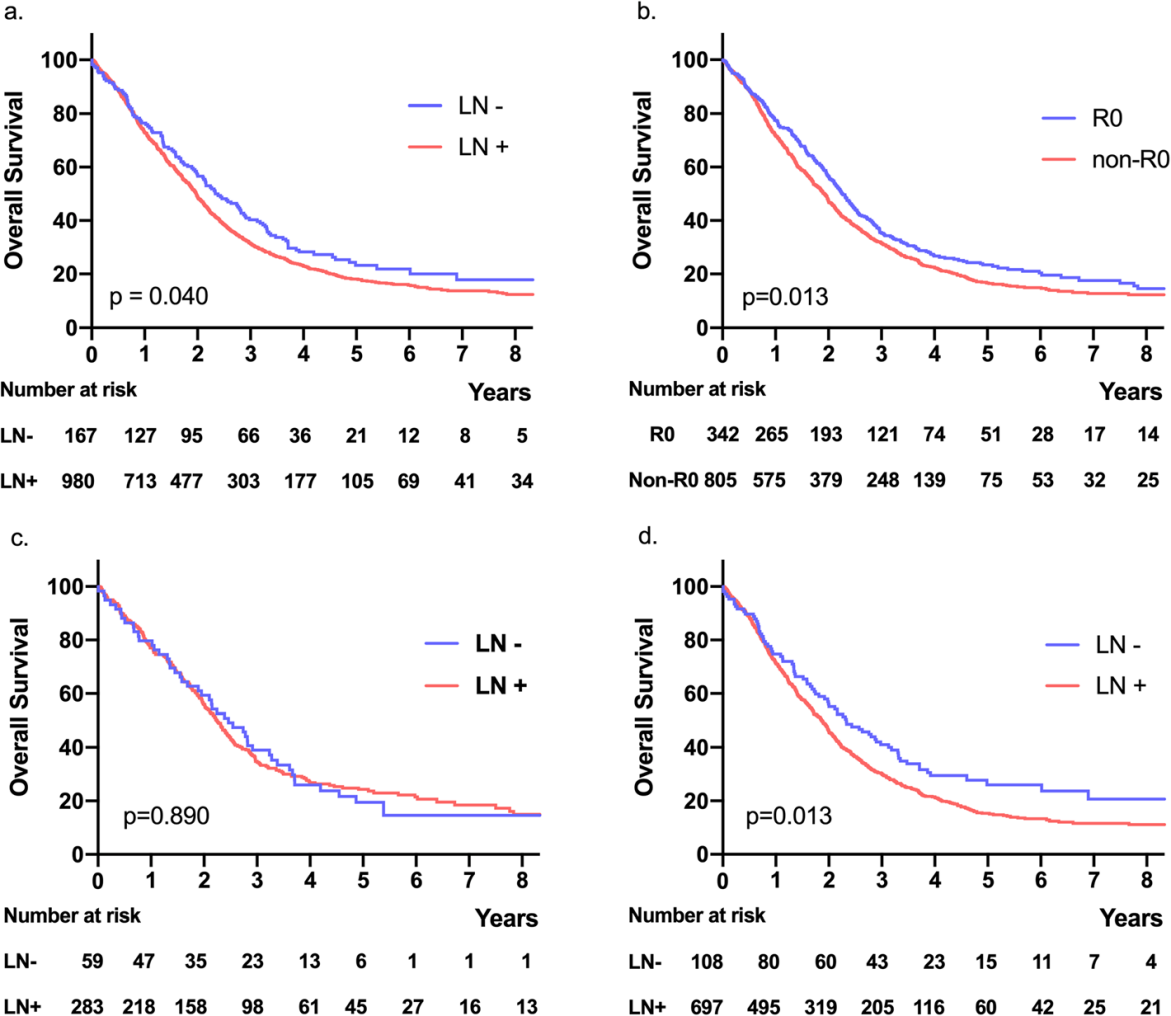
Survival analysis. **a** Five-year overall survival rate of all patients (LN− vs. LN+, p = 0.040). **b** Five-year overall survival rate of all patients (R0 vs. non-R0, p = 0.013). **c** Five-year overall survival rate of curative (R0) resection patients (LN− vs. LN+, p = 0.890). **d** 5-year overall survival rate of non-curative (non-R0) resection patients (LN− vs. LN+, p = 0.013)

The 5-year OS rate was 18.8%, with a median survival of 24 months for all patients analyzed. In the LN− group, the 5-year OS rate was 23.2%, with a median survival of 28 months. In the LN+ group, the 5-year OS rate was 18.1%, with a median survival of 23.7 months. Patients in the LN+ group had a lower 5-year OS rate than those in the LN− group (LN− vs. LN+ 23.2% vs. 18.1%; p = 0.040) (Fig. 2). Survival analysis of patients with curative (R0) and non-curative (non-R0) resection was performed (n = 1147, 342 patients in the R0 group and 805 patients in the non-R0 group). In the R0 resection group, the 5-year OS rate was 23.4%, with a median survival of 27.1 months, whereas in the non-R0 resection group, the 5-year OS rate was 16.7%, with a median survival of 23.1 months. Patients in the R0 resection group had a higher 5-year OS rate than those in the non-R0 resection group (R0 vs. non-R0 23.4% vs. 16.7%; p = 0.013) (Fig. 2).

Survival analysis of patients with curative (R0) resection of both primary and metastatic lesions was performed (n = 342, 59 patients in the LN− group and 283 patients in the LN+ group). The 5-year OS rate was 23.5%, with a median survival of 27.1 months for all R0 resection patients analyzed. In the LN− group, the 5-year OS rate was 19.5%, with a median survival of 29.5 months, whereas in the LN+ group, the 5-year OS rate was 24.3%, with a median survival of 27 months. The 5-year OS rate was not significantly different between the two groups (LN− vs. LN+ 19.5% vs. 24.3%; p = 0.890) (Fig. 2).

Survival analysis of patients with non-curative (non-R0) resection was performed (n = 805, 108 patients in the LN− group and 697 patients in the LN+ group). The 5-year OS rate was 16.7%, with a median survival of 23 months for all non-R0 resection patients analyzed. In the LN− group, the 5-year OS rate was 26%, with a median survival of 28 months, whereas in the LN+ group, the 5-year OS rate was 15.4%, with a median survival of 22.4 months. Non-R0 resection patients in the LN+ group had a lower 5-year OS rate than those in the LN− group (LN− vs. LN+ 26% vs. 15.4%; p = 0.013) (Fig. 2).

## Discussion

The main aim of this study was to examine the role of regional LNs resected during surgery in mCRC patients. The LN− group had 167 patients, while the LN+ group had 980 patients. The two groups had similar demographic data and preoperative laboratory variables. No significant difference between the two groups was found for postoperative morbidity and mortality rates. Regarding histopathologic features of primary tumor, the LN+ group exhibited a higher rate of T4 tumors and poorly differentiated adenocarcinoma. The presence of both LVI and PNI was higher in the LN+ group than that in the LN− group. Patients in the LN− group had a higher frequency of lung metastasis, whereas peritoneal seeding and systemic node metastasis occurred more frequently in the LN+ group. The 5-year OS rate of the LN+ group was significantly poorer than that of the LN− group.

In this study, the LN+ group exhibited a higher rate of poorly differentiated adenocarcinoma as well as a higher rate of LVI and PNI than that in the LN− group. The 5-year OS rate in the LN+ group was significantly poorer than that in the LN− group (LN− vs. LN+ 23.2% vs. 18.1%; p = 0.040). Our previous research suggested that poor differentiation and LVI were independent risk factors predicting LN metastasis in pT1–2 rectal carcinoma [30]. One other study also indicated that LVI and PNI were poor prognostic factors in patients with stages II and III CRC [31]. One Korean study analyzed patients with T1 or T2 CRCs who underwent radical surgery with regional lymphadenectomy, revealing that LN status was the only significant independent prognostic factor for both OS (p = 0.025) and disease-free survival (p = 0.040); moreover, the presence of LVI (p < 0.001) or PNI (p = 0.004) was an independent predictor of LN metastasis [32]. Liebig et al. also reported that PNI could serve as an independent prognostic factor in CRC patients [33]. As for mCRC, the present study’s findings imply that patients in the LN+ group had more characteristics related to poorer prognosis (poor differentiation, presence of LVI and PNI), thus, resulting in a relatively unfavorable outcome than patients in the LN− group.

Among the patients, the 5-year OS rate in the LN+ group was significantly lower than that in the LN− group (LN− vs. LN+ 23.2% vs. 18.1%; p = 0.040). The study by Berger et al. suggested that regional LN metastasis is the foremost factor in determining stage II and stage III CRC patients’ prognosis [34]. Many studies have also analyzed prognostic factors in mCRC patients and have suggested the negative prognostic impact of regional LN metastasis on OS [7, 8, 23, 24]. The 5-year survival rate in the R0 resection group was significantly better than that in the non-R0 resection group (23.4% vs. 16.7%, respectively; p = 0.013). Nevertheless, the 5-year OS rate revealed no significant difference between the LN+ and LN− groups in R0 resection patients (LN− vs. LN+ 19.5% vs. 24.3%; p = 0.890). This finding may imply that if R0 resection could be achieved, the patients in the LN+ group could have similar favorable survival outcomes as the patients in the LN− group. In other words, mCRC patients with regional LN metastasis may benefit from curative resection of primary and metastatic lesions. In recent retrospective studies, curative resection confers better long-term survival and is an independent factor for predicting better prognosis in patients with mCRC [35, 36].

Patients in the LN+ group had a lower rate of lung metastasis but a higher rate of peritoneal seeding and systemic node metastasis than that patients in the LN− group (lung metastasis in LN− vs. LN+, 25.7% vs. 14.6%, p < 0.001; peritoneal seeding in LN− vs. LN+, 17.4% vs. 32.4%, p < 0.001; systemic node metastasis in LN− vs. LN+, 6% vs. 17%, p < 0.001). These findings may be related to the CRC metastasis routes. The “seed-and-soil” hypotheses of metastatic spread in some studies suggest that metastases in the first draining site may act as seeds for further metastasis. CRC has three metastatic routes, namely, hematogenous, lymphatic, and transcoelomic/transperitoneal spread. In hematogenous spread, the metastatic sites depend on the location of the primary lesion. Blood is drained from the colon and proximal rectum through the portal system to the liver and then to the lung via the heart, as well as from the middle and distal rectum through the IVC rather than the portal vein, thus directly reaching the lung. Regarding lymphatic spread, all gastrointestinal system sites share a common lymphatic drain flowing through the cisterna chyli to the thoracic duct, then to the left subclavian vein, and finally to the lungs. In transcoelomic/transperitoneal spread, metastases spread through the peritoneal fluid in the peritoneal cavity [24, 27].

We propose two possible mechanisms resulting in more lung metastases in patients with LN− mCRC. First, patients with no regional LN involvement in malignancy would have higher lung metastasis rates due to cancer cells entering the thoracic duct directly and seeding on the lung parenchyma instead of metastasizing to the regional LNs. Second, cancer cells may have an affinity for organ-specific metastasis. Studies have suggested that CRC metastasis predominantly affects the liver due to a number of factors, including liver circulation patterns and microvessels, metastasis-related genes, chemokines and their receptors, and cellular adhesion molecules [37, 38]. We believe that at least two types of cancer cells are involved: one with more affinity to the liver and the other to the LNs and lungs. If the latter type does not cause metastasis in the LNs, they will cause lung metastasis in the first organ encountered. Adding to this complexity is the fact that LNs have their own blood supply, and thus, lymphatic drains are connected with the blood vessels, suggesting that lymphatic and hematogenous spread might transform one into the other or occur simultaneously.

Systemic LN metastases occur more frequently in patients with LN+ mCRC. This might be because more tumor cells exist in mCRC patients with regional LN metastasis, spreading through the lymphatic system and arresting at systemic LNs. As for the greater frequency of peritoneal metastases in LN+ mCRC patients, we believe that mCRC with regional LN metastasis indicates a more locally advanced disease, which might thus be accompanied by a higher incidence of transperitoneal spread.

This study has some potential limitations. First, this was a retrospective study and, thus, subject to various biases. Second, the cohort only included patients who underwent primary tumor resection with or without distant metastasectomy, which does not represent the entirety of mCRC. Some patients with severe distant metastasis who did not undergo surgical resection of the primary tumor might have had poorer outcomes. Third, the precise metastatic route of distant organs such as the liver and lungs in the LN+ group might be too intricate and complicated to distinguish because hematogenous, lymphatic, and transperitoneal spread, or the combined effect of any two or all three, are equally probable. Fourth, we did not analyze the regimen of postoperative systemic chemotherapy and target therapy in this study, which may have influence on survival. Although we shared similar treatment strategy for mCRC patients treated in this single institute, this may cause bias on OS.

In conclusion, mCRC patients with positive LNs who underwent primary tumor resection may present with high-risk pathological features, including T4 tumors, poorly differentiated adenocarcinoma, LVI, and PNI. Patients with no regional LN involvement in malignancy had a higher rate of lung metastasis, whereas those with such involvement had a higher rate of peritoneal seeding and systemic node metastasis. We confirmed that patients with surgically resected positive LNs had much poorer long-term outcomes compared with lymph node negative patients. Nevertheless, with curative resection of both primary and metastatic lesions, mCRC patients with regional lymph node metastasis could have similar survival outcomes as patients without regional lymph node metastasis.

## Data Availability

The datasets used and/or analyzed during the current study are available from the corresponding author on reasonable request.

## Abbreviations

mCRC: Metastatic colorectal cancer
CRC: Colorectal cancer
LN: Lymph node
LN−: LN-negative
LN+: LN-positive
OS: Overall survival
CEA: Carcinoembryonic antigen
LVI: Lymphovascular invasion
PNI: Perineural invasion
